# Fully Robotic Ivor-Lewis Esophagectomy Versus Hybrid Robotic Esophagectomy—A Review and Meta-Analysis of the Clinical Outcomes

**DOI:** 10.3390/jcm14248902

**Published:** 2025-12-16

**Authors:** Michele Manigrasso, Anna D’Amore, Francesco Maione, Nicola Gennarelli, Carmine Iacovazzo, Marco Milone, Pietro Anoldo

**Affiliations:** 1 “Federico II” University Hospital of Naples, Via Pansini 5, 80131 Naples, Italy; nicogenna@yahoo.it (N.G.); pietro.anoldo@gmail.com (P.A.); 2 Department of Clinical Medicine and Surgery, “Federico II” University of Naples, Via Pansini 5, 80131 Naples, Italy; anna.damore.md@gmail.com (A.D.); francescomaione79@gmail.com (F.M.); milone.marco.md@gmail.com (M.M.); 3 Department of Neuroscience, Reproductive Science and Odontostomatological Science, “Federico II” University of Naples, Via Pansini 5, 80131 Naples, Italy; carmine.iacovazzo@unina.it

**Keywords:** esophagectomy, robotic, hybrid, cancer

## Abstract

**Background:** Esophageal cancer ranks among the top ten most prevalent cancers worldwide and remains a significant contributor to cancer-related mortality. While surgery combined with neoadjuvant therapy stands as the cornerstone treatment, the evolution of surgical techniques towards minimally invasive procedures has shown promising results. Robotic Assisted Minimally Invasive Esophagectomy (RAMIE) emerges as a potential advancement, offering precise movements and a three-dimensional endoscopic view. Against this backdrop, clarifying whether a fully robotic approach provides measurable perioperative or early oncologic advantages over a hybrid technique is clinically relevant. Despite initial skepticism, studies comparing fully robotic and hybrid approaches for esophagectomy have been conducted to evaluate their feasibility and sustainability. **Methods:** A systematic review and meta-analysis were performed following PRISMA guidelines. Four retrospective studies comparing fully robotic and hybrid approaches were included, comprising 1540 patients. **Results:** Intraoperative outcomes favored the fully robotic approach, showing shorter operative times and reduced blood loss (MD = −41 min, *p* = 0.056, 95% CI: −83.202; 0.994 and MD = −48.762 mL, *p* = 0.040, 95% CI: −95.257; −2.266, respectively). Additionally, the fully robotic approach demonstrated advantages in terms of lymph node retrieval and shorter ICU and hospital stay (MD = −0.894, *p* < 0.0001, 95% CI: −1.224; −0.564, MD = −1.139 days, *p* < 0.0001, 95% CI: −1.313; −0.965 and MD = −3.264 days, *p* = 0.011, 95% CI: −5.767; −0.760, respectively). **Conclusions:** Although limitations exist, including the retrospective nature of the studies and limited sample size, the findings suggest that the fully robotic approach may offer superior outcomes compared to the hybrid approach for Ivor-Lewis esophagectomy. These results highlight the potential of robotics in enhancing safety and effectiveness in oesophageal cancer surgery, encouraging further consideration and adoption by surgeons.

## 1. Introduction

Esophageal cancer ranks among the most lethal solid tumors worldwide, currently the eighth most prevalent malignancy and the sixth leading cause of cancer-related deaths, with a disproportionately high burden in certain regions of Asia and Africa compared with Western countries [1]. Over the last two decades, a multidisciplinary paradigm has become the rule rather than the exception: combinations of chemotherapy, chemoradiation, and tailored surgery have yielded meaningful improvements in survival for selected patients, especially after the adoption of protocols such as CROSS, which established the benefit of neoadjuvant chemoradiotherapy followed by resection for resectable disease of the esophagus and esophagogastric junction [2]. While a multidisciplinary approach is crucial in managing this cancer, surgery coupled with neoadjuvant therapy remains the cornerstone of its treatment [2,3,4].

The evolution of surgical techniques for oesophageal cancer mirrors advancements in other abdominal and thoracic malignancies. After decades in which open esophagectomy represented the standard, minimally invasive techniques emerged to reduce surgical trauma and enhance recovery while preserving oncologic radicality. The benefits of minimally invasive esophagectomy (MIE) reported in randomized and observational cohorts include lower rates of pulmonary complications, reduced estimated blood loss, decreased wound morbidity, and shorter convalescence, without compromising resection quality [5,6,7]. However, despite these advantages, minimally invasive esophagectomy has not yet attained the status of standard care, likely due to technical challenges associated with this approach.

In the realm of surgical evolution, the robotic approach appears promising in overcoming certain challenges raised up by laparoscopy [8,9,10,11]. Robotic technology offers a three-dimensional endoscopic view and utilizes the EndoWrist^®^ technology, enabling precise movements in confined spaces.

There is global enthusiasm for Robotic Assisted Minimally Invasive Esophagectomy (RAMIE). However, skepticism persists regarding the benefits of this approach, particularly following reports indicating minimal advantages of robotic assistance during the abdominal phase [12,13]. Consequently, real-world multicenter experiences and registry-based studies have, in parallel, documented heterogeneous practices, with many teams adopting hybrid strategies—laparoscopy in the abdomen plus robotic transthoracic esophagectomy—while others perform a fully robotic procedure throughout.

Against this backdrop, clarifying whether a fully robotic approach provides measurable perioperative or early oncologic advantages over a hybrid technique is clinically relevant. This is particularly true for systems-level decision making (resource allocation, operating room scheduling, and training) and for patient counseling. The present systematic review and meta-analysis were designed to compare fully robotic versus hybrid Ivor-Lewis esophagectomy for esophageal cancer, with the working hypothesis that robotic assistance across both phases could be feasible, safe, and potentially advantageous relative to a hybrid approach in carefully selected patients. Our analysis sought to appraise intraoperative parameters (operative time, blood loss, conversions), postoperative complications (including anastomotic leakage, pneumonia, and mortality), oncologic surrogates (R0 resection and lymph node yield), and recovery metrics (ICU and hospital length of stay).

## 2. Materials and Methods

### 2.1. Literature Search and Study Selection

This systematic review adhered to the reporting guidelines outlined in the PRISMA (Preferred Reporting Items for Systematic Reviews and Meta-Analyses) (Supplementary Materials) [14] and aligned with the Meta-Analysis of Observational Studies in Epidemiology (MOOSE) guidelines [15]. These frameworks informed the formulation of the research question, the structuring of eligibility criteria, the design of the search strategy, and the transparent reporting of study selection, data extraction, and synthesis. The risk-of-bias assessment instruments were prespecified, and analytical choices—including random-effects modeling and heterogeneity metrics—were defined a priori to reduce analytical flexibility and enhance reproducibility.

The search strategy employed the string “hybrid AND (oesophagus OR esophagus OR esophagectomy OR oesophagectomy)” across the online databases Cochrane, EMBASE, PubMed, SCOPUS, and Web of Science. Only articles in written English were considered. Abstracts of posters and podium presentations from international meetings, systematic reviews, and meta-analyses were excluded. Additionally, the reference lists of retrieved studies were screened.

The research question was formulated using a PICO (Problem/Population, Intervention, Comparison, Outcome) framework. The population consisted of patients diagnosed with oesophageal cancer. The intervention under consideration was Ivor-Lewis esophagectomy, comparing fully robotic procedures versus a hybrid approach involving laparoscopic abdominal techniques and robotic transthoracic esophagectomy.

The outcomes assessed encompassed intraoperative measures (operative time, mean blood loss, overall conversions, and conversions specifically during the abdominal phase), postoperative complications (such as anastomotic leakage, pneumonia, mortality), oncologic indicators (harvested nodes, R0 resection rate), and recovery metrics (Intensive Care Unit stay and length of hospital stay).

Two independent reviewers conducted the search and study selection. Any discrepancies were resolved through consultation with a third investigator, facilitating consensus to achieve agreement.

Data extraction and risk of bias assessment were performed. In addition to the mentioned outcomes, the following data were extracted from each study: primary author, year of publication, study design, sample size, gender distribution, mean age, mean BMI (Body Mass Index), ASA (American Society of Anesthesiologists) Score, tumor stage based on UICC (Union for International Cancer Control) criteria, and the rate of preoperative radio-chemotherapy.

Observational study quality was assessed via the Newcastle–Ottawa Scale (NOS), which evaluates selection, comparability, and outcome domains to yield a score from 0 to 9 [16]. For completeness, we prespecified the Cochrane Collaboration tool for randomized trials, although no eligible RCTs were identified in this topic area [17].

### 2.2. Statistical Analysis

The statistical analysis was conducted using Comprehensive Meta-Analysis [Version 2.2, Biostat Inc., Englewood, NJ, USA, 2005].

For dichotomous outcomes, odds ratios (ORs) with 95% confidence intervals (CIs) were adopted. In situations involving rare events, the risk difference (RD) along with corresponding 95% CI was utilized, following the approach outlined by Messori et al. [18]. Studies that reported median, range, and sample size or those reporting median and quartile ranges had their means and standard deviations estimated using methodologies by Shi, Luo, and Wan [19,20,21]. If studies presented mean values without standard deviations, these were imputed based on the methods by Furukawa et al. [22].

Z-scores were employed to determine the overall effect, with significance set at *p* < 0.05.

The summary estimate was calculated assuming a random effects model, following the approach by DerSimonian and Laird [23]. Heterogeneity among the studies was quantified using the I^2^ statistic, where values <25% indicated low heterogeneity, values between 25% and 50% indicated moderate heterogeneity, and values >50% indicated high heterogeneity [24,25].

Publication bias was evaluated using a visual assessment of funnel plot symmetry and was further tested via Egger’s linear regression method [26]. A significance level of *p* < 0.05 was considered indicative of publication bias.

## 3. Results

### 3.1. Study Selection

**Figure 1:**
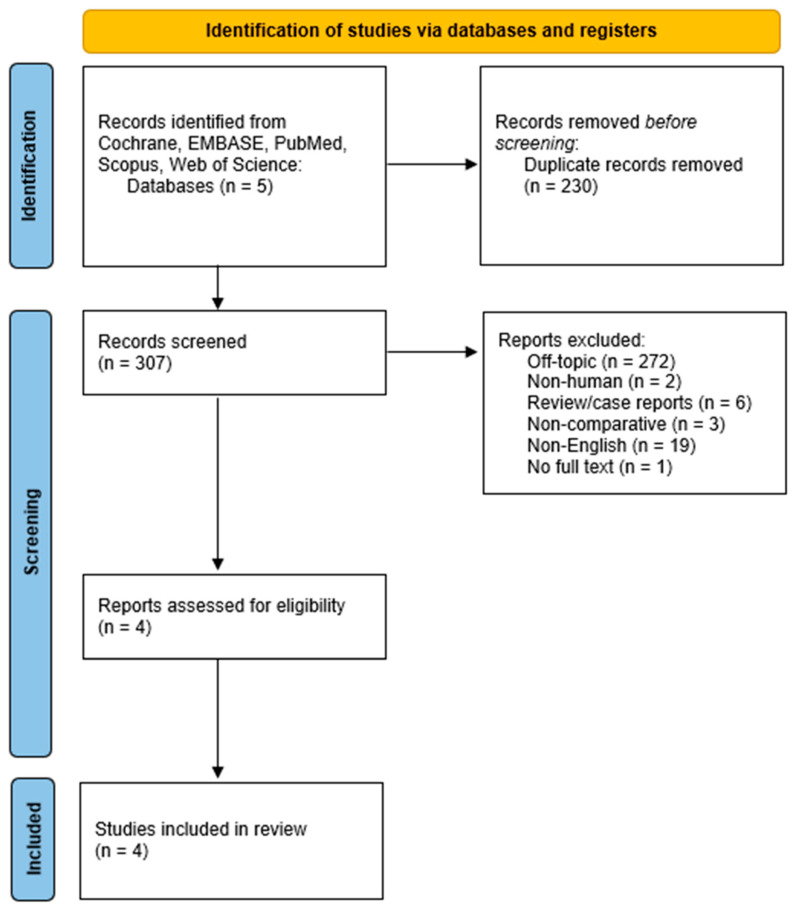
PRISMA flowchart of the include studies.

The literature search initially identified 537 articles. Upon eliminating duplicates, 307 unique articles underwent analysis. Among these, 303 articles were excluded due to being off-topic (272), pertaining to non-human subjects (2), classified as reviews (1), comprising case reports/series (5), lacking comparability (non-comparative studies) (3), lacking full-text availability (1), or not being in the English language (19). Consequently, the final analysis encompassed 4 articles that directly compared the fully robotic approach to the hybrid approach in Ivor-Lewis esophagectomy for oesophageal cancer [3,13,27,28]. The process of study selection is visually depicted in the PRISMA flowchart (Figure 1).

### 3.2. Study Characteristics

All included studies were retrospective comparative analyses [3,13,27,28]. Among these, three were multicentric [3,13,28], while one was monocentric [27]. The cumulative sample comprised 1540 patients—907 treated with a fully robotic approach and 633 treated with a hybrid approach. Baseline characteristics, where reported, suggested broadly comparable case-mixes with respect to age, sex, BMI, ASA class, tumor stage, and rates of neoadjuvant chemoradiation. One of the multicenter series incorporated propensity-score techniques to improve comparability, whereas others relied on conventional multivariable adjustment or crude comparisons, reflecting real-world practice heterogeneity. The summarized characteristics of the included studies are presented in Table 1.

### 3.3. Risk of Bias Assessment

All studies obtained NOS quality scores higher than 6, signifying a fair methodological quality. Specifically, three studies attained an NOS quality score of 7 [3,27,28], while one study achieved an NOS quality score of 8 [13]. The detailed NOS quality scores are presented in Supplementary Table S1. Notably, the analysis did not include any Randomized Controlled Trials (RCTs) comparing robotic and hybrid esophagectomy.

### 3.4. Intraoperative Outcomes

**Figure 2:**
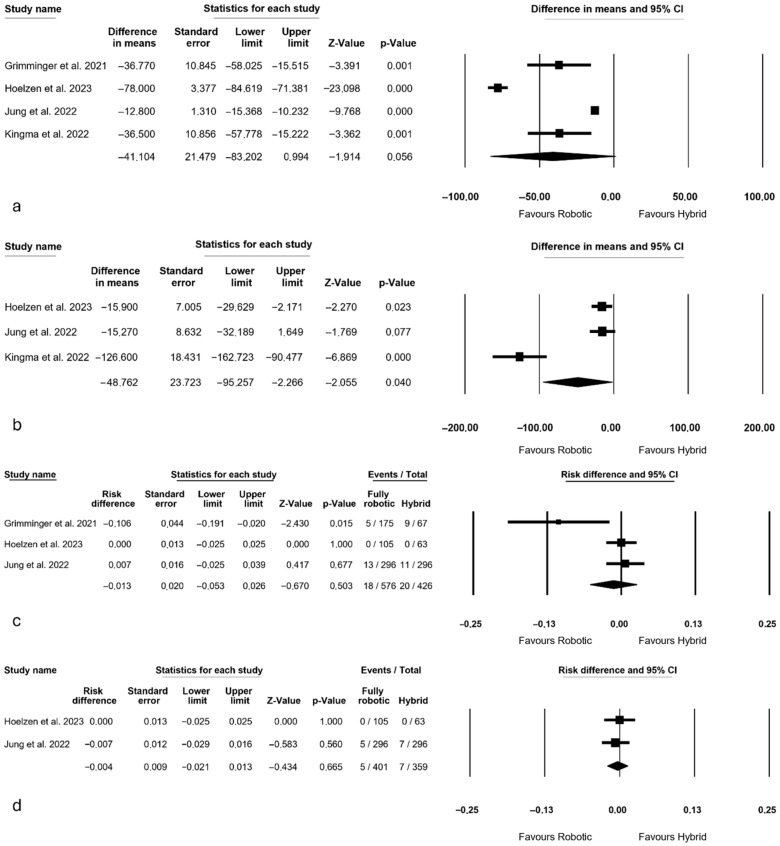
Intraoperative outcomes: (**a**) mean operative time [3,13,27,28]; (**b**) mean blood loss [3,27,28]; (**c**) overall conversions [13,27,28]; (**d**) conversion during the abdominal phase [27,28].

Intraoperative outcomes are depicted in Figure 2. All authors [3,13,27,28] reported operative time, revealing a trend towards a shorter operative duration in the robotic group (MD = −41 min, *p* = 0.056, 95% CI: −83.202; 0.994). There was a notable and significant heterogeneity among the studies (I^2^ = 98.115%, *p* < 0.0001).

Regarding mean blood loss, three authors conducted analyses [3,27,28], indicating that a fully robotic approach correlated with reduced mean blood loss (MD = −48.762 mL, *p* = 0.040, 95% CI: −95.257; −2.266), with substantial heterogeneity observed among the studies (I^2^ = 86.050%, *p* = 0.001).

Three authors reported the number of conversions [13,27,28], demonstrating similar values between the two groups (RD = −0.013, *p* = 0.503, 95% CI: −0.053; 0.026). However, there was significant heterogeneity among these studies (I^2^ = 66.617%, *p* = 0.050). Hoelzen et al. and Jung et al. [27,28] also reported the number of conversions occurring during the abdominal phase, showing no significant difference between the two approaches (RD = −0.004, *p* = 0.665, 95% CI: −0.021; 0.013), with no heterogeneity observed between these two studies (I^2^ = 0%, *p* = 0.696)

### 3.5. Postoperative Complications

**Figure 3:**
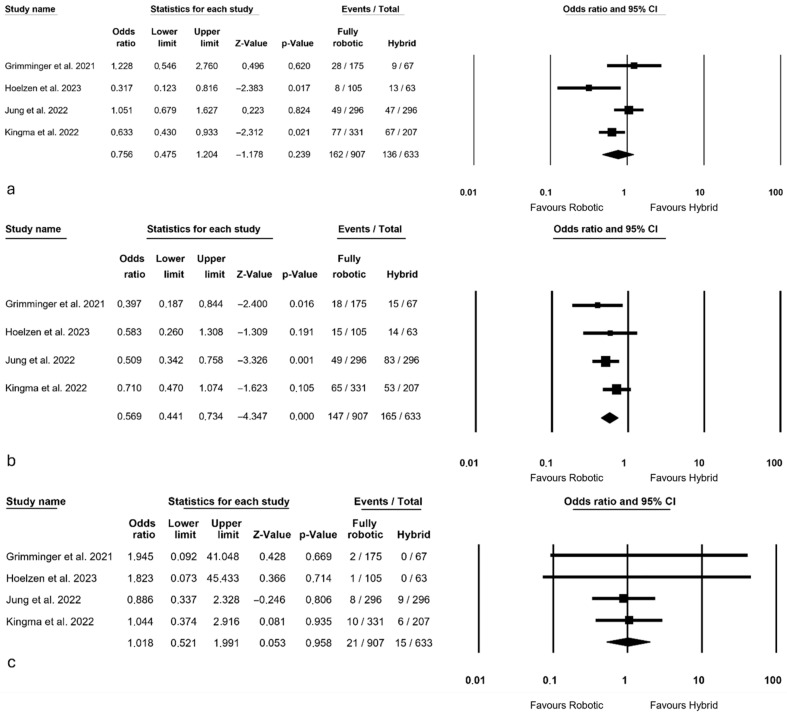
Postoperative complications: (**a**) pneumonia; (**b**) anastomotic leakage; (**c**) mortality [3,13,27,28].

Postoperative complications are presented in Figure 3. Regarding pneumonia, analysis by all authors [3,13,27,28] resulted in a similar number of postoperative pneumonias between the two groups (OR = 0.756, *p* = 0.239, 95% CI: 0.475; 1.204), with considerable heterogeneity observed among the studies (I^2^ = 60.431%, *p* = 0.055).

All authors reported on anastomotic leakage [3,13,27,28], revealing a significant difference in favor of the robotic approach (OR = 0.569, *p* < 0.0001, 95% CI: 0.441; 0.734) with no significant heterogeneity among the studies (I^2^ = 0%, *p* = 0.517).

Mortality, reported by all authors [3,13,27,28], showed no differences between the two groups (OR = 1.018, *p* = 0.958, 95% CI: 0.521; 1.991) and no heterogeneity among the studies (I^2^ = 0%, *p* = 0.944).

### 3.6. Oncologic Outcomes

**Figure 4:**
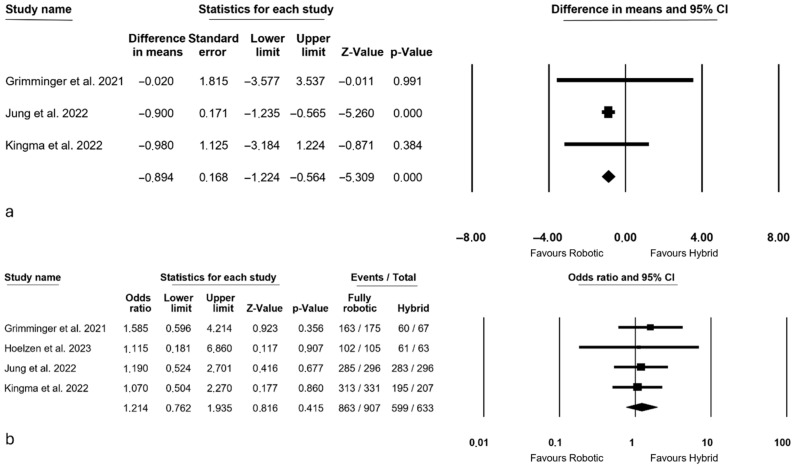
Oncologic outcomes: (**a**) number of harvested nodes [3,13,28]; (**b**) R0 resections [3,13,27,28].

Oncologic outcomes are presented in Figure 4. The mean number of harvested nodes was reported by 3 authors [3,13,28], indicating a higher count of retrieved nodes in the fully robotic group (MD = −0.894, *p* < 0.0001, 95% CI: −1.224; −0.564), with no observed heterogeneity among the studies (I^2^ = 0%, *p* = 0.887).

The assessment of the number of R0 resections, conducted by all authors [3,13,27,28], demonstrated comparable values between the two groups (OR = 1.214, *p* = 0.415, 95% CI: 0.762; 1.935), with no heterogeneity observed among the studies (I^2^ = 0%, *p* = 0.939).

### 3.7. Recovery Outcomes

**Figure 5:**
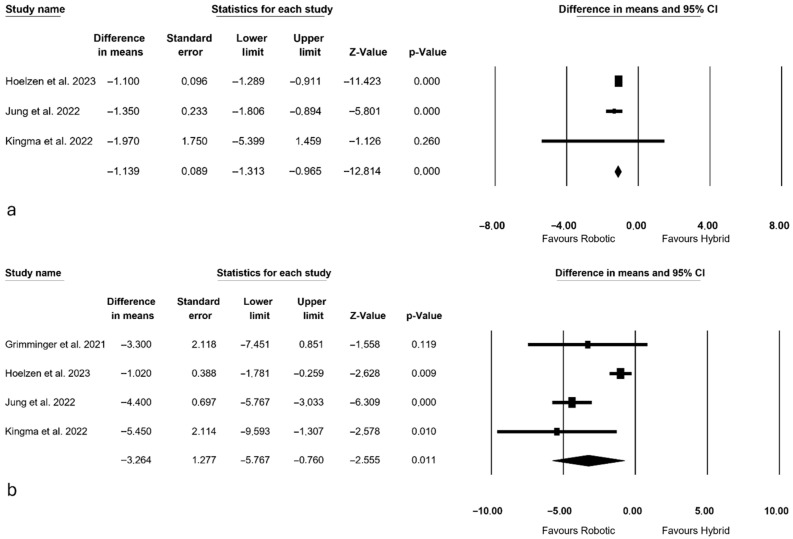
Recovery outcomes: (**a**) ICU length of stay [3,27,28]; (**b**) hospital length of stay [3,13,27,28].

Recovery outcomes were depicted in Figure 5. ICU stay was reported by 3 authors [3,27,28], revealing a significantly shorter duration in the fully robotic group (MD = −1.139 days, *p* < 0.0001, 95% CI: −1.313; −0.965), albeit with a high level of heterogeneity observed among the studies (I^2^ = 97.100%, *p* < 0.0001).

The length of hospital stay was analyzed by all authors [3,13,27,28], demonstrating a significantly shorter duration in the fully robotic group (MD = −3.264 days, *p* = 0.011, 95% CI: −5.767; −0.760), with a moderate level of heterogeneity among the studies (I^2^ = 55.942%, *p* = 0.078).

### 3.8. Publication Bias

The visual funnel plots exhibited symmetry across most analyzed outcomes, and the Egger’s test did not indicate any suggestion of publication bias, except for the mean number of harvested nodes and operative time. In these cases, visual inspection suggested an asymmetric distribution of studies around the mean, and the Egger’s test confirmed significant publication bias (*p* = 0.01 and *p* = 0.006, respectively).

### 3.9. Assessment of Certainty

The assessment of certainty of the analyzed outcomes is depicted in Table 2.

## 4. Discussion

The adoption of minimally invasive esophagectomy (MIE) has gained considerable traction since its introduction in the early 2000s [6,7,8]. The advantages of the minimally invasive approach over traditional surgery, well-documented in the literature, encompass improved quality of life [29], reduced total complication rates, decreased intraoperative blood loss [30], lower incidences of wound infection and pulmonary complications [31], as well as decreased in-hospital mortality and morbidity [32], and enhanced postoperative recovery outcomes, without compromising oncological quality [33].

However, despite these recognized advantages, the adoption of MIE has yet to be universally embraced as the gold standard approach for oesophageal cancer, potentially due to the technical complexities associated with this technique.

The robotic approach has addressed several challenges linked to conventional laparoscopy. Nevertheless, the specific advantages of robotic-assisted minimally invasive esophagectomy (RAMIE) remain a subject of ongoing evaluation. Recent evidence suggests the superiority of RAMIE over MIE concerning thoracic lymph node retrieval and the incidence of postoperative pneumonia [8,9,10,11]. However, there remains limited understanding regarding the advantages of the robotic approach during the abdominal phase of the procedure [12].

Recent research has suggested that the robotic approach might be unnecessary during the abdominal phase of Ivor-Lewis esophagectomy, as no significant differences in short-term or oncologic outcomes were observed compared to laparoscopy [12]. As a result, the hybrid approach continues to be favored in current practice.

The objective of our meta-analysis is to compare the hybrid and fully robotic approaches to Ivor-Lewis esophagectomy to ascertain whether the latter may be deemed unnecessary for this intervention. To the best of our knowledge, this meta-analysis is the first of its kind, and the findings obtained are very promising.

The intraoperative findings affirm the safety of the fully robotic approach, demonstrating no significant difference in reported conversion rates, even during the abdominal phase. Conversions typically reflect either oncotechnical challenges (dense adhesions, bleeding) or patient factors (body habitus), which may not be fully alleviated by robotics alone.

While the reduced blood loss observed in the fully robotic group may reasonably be attributed to the greater precision of robotic instrument movements, the shorter operative time should be interpreted with caution. This difference may be incidental and related to surgeon expertise rather than an intrinsic advantage of the robotic platform. Indeed, the current literature generally reports shorter operative times for laparoscopic procedures compared with robotic surgery.

Similarly, postoperative complications favor the fully robotic approach.

For pneumonia, heterogeneity in perioperative care (e.g., analgesia, physiotherapy, ventilatory strategies, and early mobilization) likely dominates over subtle differences in surgical approach [5,6,7]. Standardized enhanced recovery pathways and robust respiratory bundles may exert effects larger than any incremental benefit from robotic assistance during the abdominal phase, potentially explaining the null finding with significant between-study variability.

Interestingly, the fully robotic approach correlated with a reduced number of anastomotic leakages. Although the intrathoracic anastomosis is constructed during the thoracic phase in Ivor-Lewis procedures, conduit quality depends critically on abdominal phase steps—gentle gastric mobilization, preservation of the right gastroepiploic arcade, and avoidance of traction or devascularization—each of which may benefit from enhanced dexterity and visualization. However, we cannot exclude that the lower leakage rate in the fully robotic group may be influenced by surgeon experience, anastomosis fashion, patient selection, or other unmeasured confounders, rather than a direct intrinsic advantage of the robotic abdominal phase.

Moreover, the fully robotic approach was associated with a higher number of harvested lymph nodes, even if this finding should be interpreted with caution. The available studies did not report lymph-node yield separately for the abdominal and thoracic phases, and since the thoracic phase was performed robotically in both groups, this difference cannot be attributed to the thoracic dissection. A plausible explanation is that the robotic platform may enhance the precision and thoroughness of the abdominal lymph-node dissection compared with conventional laparoscopy. However, this interpretation remains speculative due to limited phase-specific data. The similar R0 rates reassure that oncologic adequacy is preserved regardless of the abdominal instrument set, provided the thoracic component is robotic and the case is undertaken by experienced teams.

Lastly, the noteworthy outcomes of the fully robotic approach are the reduced lengths of postoperative ICU and hospital stays. These results could be linked to the shorter operative times and the aforementioned superior intraoperative and postoperative outcomes.

Despite the promising results of this meta-analysis, certain limitations should be acknowledged.

The observational design of all included studies necessitates caution about residual confounding despite careful statistical adjustments in some cohorts [13,28]. Variables such as surgeon experience, learning curve stage, center volume, and perioperative protocols can influence outcomes yet are difficult to fully control in retrospective analyses [16,24,25]. Our use of random-effects modeling acknowledges such diversity and appropriately widens CIs to reflect between-study variability [23,24,25]. Data harmonization posed challenges for a subset of continuous outcomes requiring transformations from medians and ranges, which we addressed with validated methods (Shi/Luo/Wan) and SD imputation when necessary (Furukawa) [19,20,21,22]. Signals of small-study effects for operative time and nodal yield remind us that publication bias cannot be excluded, and additional large-scale, multicenter data are needed to refine estimates [26].

From a practical standpoint, the results support the feasibility and potential advantages of a fully robotic Ivor-Lewis esophagectomy in experienced centers. The reduction in anastomotic leaks is clinically meaningful given the morbidity, resource utilization, and potential survival impact of this complication. Even modest reductions in intraoperative blood loss and shorter ICU/hospital stays can translate into tangible benefits for patients and health systems, particularly within enhanced recovery programs [5,6,7,29]. That said, infrastructure requirements, robot access, and team training remain pivotal considerations. Institutions with mature hybrid programs achieving excellent outcomes may prioritize consistency and volume over wholesale conversion to full robotics, whereas centers building RAMIE pathways can reasonably consider a fully robotic configuration from the outset [3,8,9,10,11,12].

Several knowledge gaps warrant attention. First, prospective, ideally randomized, studies comparing fully robotic and hybrid strategies would provide more definitive evidence free from unmeasured confounding [17]. Second, patient-reported outcomes (quality of life, symptom burden), already shown to improve with MIE over open surgery, should be systematically measured to detect any incremental benefit of full robotics over hybrid approaches [29]. Third, cost-effectiveness analyses incorporating device amortization, operating room throughput, complication-related costs (particularly leaks), and LOS are essential for health system decision making. Fourth, long-term oncologic endpoints (disease-free and overall survival) and functional outcomes (dysphagia relief, nutritional status) should be assessed to understand whether perioperative gains translate into durable benefits [5,6,7,9,10,11,29,30,31,32,33]. Finally, structured training and proctoring frameworks, potentially leveraging registry infrastructures, may shorten learning curves and disseminate best practices for RAMIE [3,10,11,13].

Strengths include comprehensive multidatabase searching, duplicate independent screening and extraction, prespecified analytical choices aligned with PRISMA/MOOSE, use of validated transformation methods for non-normally reported data, and coherent assessment of heterogeneity and publication bias [14,15,16,17,18,19,20,21,22,23,24,25,26]. Limitations reflect those of the underlying literature: all studies were retrospective; selection bias and confounding by indication cannot be excluded; definitions of complications (e.g., pneumonia, anastomotic leak) varied; and generalizability may be limited because most included cohorts originated from Western centers with advanced minimally invasive programs [3,13,27,28]. Additionally, the small number of studies constrains the interpretability of subgroup and sensitivity analyses and increases uncertainty around point estimates in some outcomes [23,24,25,26].

Taking the body of evidence together, our meta-analysis suggests that fully robotic Ivor-Lewis esophagectomy is feasible, safe, and potentially advantageous relative to a hybrid approach, with specific benefits in anastomotic leak reduction, modest decreases in blood loss, and shorter ICU and hospital stays, while maintaining oncologic adequacy (R0 rates). Although pneumonia and mortality did not differ significantly, the directionality of several perioperative outcomes favors full robotics. Decisions at the programmatic level should integrate these findings with local expertise, case volumes, and resource considerations, recognizing that team experience and standardized perioperative care are critical determinants of outcome regardless of instrument set [3,5,6,7,8,9,10,11,12,13,27,28,29,30,31,32,33].

In conclusion, in this systematic review and meta-analysis of 1540 patients across four retrospective comparative studies, fully robotic Ivor-Lewis esophagectomy demonstrated comparable safety to hybrid esophagectomy and potential advantages in anastomotic leak, intraoperative blood loss, and postoperative recovery metrics, with no compromise in R0 resection rates and a higher lymph node yield [3,13,27,28]. While the evidence base is observational and heterogeneous, these findings support the feasibility and clinical sustainability of the fully robotic technique in experienced centers. Future prospective comparative studies—including randomized trials where feasible—should evaluate patient-centered outcomes, long-term oncologic results, and cost-effectiveness to inform definitive practice recommendations.

## Figures and Tables

**Table 1:** Patients’ characteristics of the included studies.

		Fully RAMIE										Hybrid RAMIE									
Study	Study Design					ASA (%)										ASA (%)					
		*Patients*	*Male (%)*	*Age*	*BMI*	*I*	*II*	*III*	*IV*	*Preop chemio*	*preop chemo-radio*	*Patients*	*Male (%)*	*Age*	*BMI*	*I*	*II*	*III*	*IV*	*Preop chemio*	*preop chemo-radio*
Grimminger et al. 2021 [13]	retro	175	84.6	60.46 ± 11.89	25.46 ± 5.57					43.4	38.8	67	79.1	63.78 ± 7.01	25.5 ± 5.3					35.8	49.3
Hoelzen et al. 2023 [27]	retro	105	84							29.5	61.9	63	84.1							38.1	49.2
Jung et al. 2022 [28]	retro	296	82.8	64.2 ± 1.6	26.2 ± 0.75	59.1	33.1	0.3	0	1	25	296	79.1	64.5 ± 1.49	25.66 ± 0.67	3	59.8	36.5	0.7	16.6	74
Kingma et al. 2022 [3]	retro	331										207									

BMI: Body Mass Index; RAMIE: Robot-Assisted Minimally Invasive Esophagectomy; Continuous variables are expressed in mean ± SD and categorical variables are expressed in percentages. Patients are expressed as numbers.

**Table 2:** GRADE table of the analyzed outcomes. ⬤ = level of certainty present; ◯ = level of certainty absent. The number of ⬤ reflects the overall certainty of evidence (from low to high).

Outcome	Effect (OR/RD/MD)	Number of Patients (Studies)	Certainty (GRADE)	Comments
Anastomotic leakage	OR ≈ 0.75 (not significant)	1540 patients (all studies)	Moderate ⬤⬤⬤◯	Consistent reduction; downgraded for observational design.
Pneumonia	OR ≈ 0.24 (not significant)	1540 patients (all studies)	Low ⬤⬤◯◯	Variability and imprecision across studies.
Intraoperative blood loss	MD ≈ –48 mL (favors fully robotic)	3 studies	High ⬤⬤⬤⬤	Objective and consistent across studies.
Operative time	MD ≈ −41 min (not significant)	4 studies	Low ⬤⬤◯◯	High heterogeneity; learning curve effects.
Lymph node yield	MD ≈ +1 (not significant)	3 studies	Moderate ⬤⬤⬤◯	Phase-specific data unavailable.
R0 resection	OR ≈ 1.21 (not significant)	All studies	High ⬤⬤⬤⬤	Objective outcome; consistent.
Mortality	OR ≈ 1.02 (not significant)	1540 patients (all studies)	High ⬤⬤⬤⬤	Objective outcome; consistent.
ICU stay	MD ≈ −1 day (favors Fully robotic)	3 studies	Moderate ⬤⬤⬤◯	Center heterogeneity
Hospital stay	MD ≈ −3 days (favors Fully robotic)	All studies	Moderate ⬤⬤⬤◯	Clinically relevant; downgraded for confounding.

## Data Availability

No new data were created or analyzed in this study.

## Supplementary Materials

The following supporting information can be downloaded at: https://www.mdpi.com/article/10.3390/jcm14248902/s1, Supplementary Table S1. NOS quality assessment of the included studies; PRISMA checklist. Reference [34] is cited in the Supplementary Materials.
