# Supplementary material for: Fully Robotic Ivor-Lewis Esophagectomy Versus Hybrid Robotic Esophagectomy—A Review and Meta-Analysis of the Clinical Outcomes
Source: J Clin Med. 2025 Dec 16;14(24):8902. doi: 10.3390/jcm14248902 (PMC12733808; doi:10.3390/jcm14248902)
Supplement: Supplementary file 1 [file jcm-14-08902-s001.zip › Table S1.pdf]

Supplementary Table S1. NOS quality assessment of the included studies

Study	Selection				Comparability	Exposure			Total
	1	2	3	4	1	1	2	3	
Grimminger et al. 2021	★	★		★	★★	★	★	★	8
Hoelzen et al. 2023	★	★		★	★	★	★	★	7
Jung et al. 2022	★	★		★	★	★	★	★	7
Kingma et al. 2022	★	★		★	★	★	★	★	7
